# Circumferential Vaginal Tear During the Cesarean Section: A Complication of Vaginal Hand Assistance in a Deeply Impacted Fetal Head

**DOI:** 10.7759/cureus.61869

**Published:** 2024-06-07

**Authors:** Anum Aziz, Rozilla S Khan

**Affiliations:** 1 Obstetrics and Gynaecology, Aga Khan University, Karachi, PAK

**Keywords:** trauma in cesarean section, second stage cesarean section, circumferential vaginal tear, reverse breech extraction, impacted fetal head

## Abstract

We present the case of a 29-year-old, G2P1+0 pregnant woman who was unbooked and presented to the emergency room at 36+5 weeks gestation with complaints of leaking liquor, labour pains, vaginal bleeding and raised blood pressure. Her history revealed previous vaginal delivery and index pregnancy complicated with obstetric cholestasis, pre-eclampsia, and fetal growth restriction. During her hospital course, the patient underwent an emergency cesarean section due to uncontrolled blood pressure and pathological cardiotocograph (CTG) revealing a deeply impacted fetal head intraoperatively and necessitating an inverted T incision on the uterus. Although the newborn was delivered successfully, a full-thickness circumferential tear in the vaginal vault was discovered, requiring immediate surgical repair with the involvement of a urologist. The patient experienced postoperative complications related to pre-eclampsia and sepsis but was eventually discharged in stable condition. This case highlights the importance of prompt diagnosis and management of obstetric emergencies especially in the case of deeply impacted fetal head, and the need for a multidisciplinary approach to address complications such as vaginal tears during cesarean sections due to vaginal assistance in delivering the baby.

## Introduction

Cesarean delivery is a frequently performed surgical intervention to deliver babies in cases of obstetric emergencies. While it is generally considered a safe procedure, second-stage cesarean sections can be associated with various complications, including extended tears, organ injury, haemorrhage or deeply impacted fetal head [1]. Impacted fetal head poses a challenge encountered in about 16% of cases requiring cesarean section due to emergency indications in the late first or second stage of labour. This condition arises when the baby's head is deeply situated in the pelvis, leading to difficulties in the delivery process [2]. In this case report, we describe a unique and rare complication - a circumferential vaginal tear in a patient who presented with multiple risk factors, including pre-eclampsia and fetal growth restriction and underwent emergency cesarean delivery due to compromised fetal well-being.

## Case presentation

A 29-year-old woman presented in her second pregnancy as an un-booked case at 36+5 weeks gestation, with previous vaginal delivery three years back. She reported complaints of leaking liquor, labour pains, and vaginal bleeding for one day. Additionally, she was known to have been diagnosed with pre-eclampsia and obstetric cholestasis during the index pregnancy. Hand-held records from the previous hospital indicated that she previously gave birth to a baby girl at 36 weeks of gestation, weighing 1.6 kg. The child is currently doing well. An ultrasound performed at 35 weeks of gestation during this pregnancy showed fetal biometric parameters corresponding to 31 weeks of gestation, an amniotic fluid index of 9.1 cm, and the placenta located at the posterior fundal region. However, obstetric Dopplers for the fetal umbilical artery and middle cerebral artery were not performed. A past medication review revealed that she had been taking ursodeoxycholic acid at a dosage of 500 mg twice a day for the past two weeks due to elevated serum total bile acids. She received steroid coverage for lung maturity one week ago.

Upon initial examination, the patient appeared apprehensive and was experiencing labour pains. A general physical examination showed signs of mild pedal oedema with pitting. Vital signs revealed elevated blood pressure (154/104 mmHg) and a heart rate of 90 beats per minute with normal breathing rate. Chest auscultation revealed bilateral normal vesicular breathing. However, clonus was negative and there were no other prodromal symptoms. Abdominal examination showed a fundal height of 32 weeks, with a longitudinal lie and 3/5th palpable cephalic presentation. Uterine contractions were palpable with frequency of 3 in 10 minutes, each lasting for 25-30 seconds. Fetal heart sounds were audible, and vaginal examination showed cervical dilation of 4 cm and mild bleeding, presenting part was at station of -2. The patient was admitted and shifted to the labour room after administration of intravenous labetalol 20 mg and a loading dose of magnesium sulphate 4 grams. Magnesium sulphate was given due to persistently raised blood pressure. Based on the maternal condition, the family was counselled for admission and the patient was shifted to the labour and delivery suite. Investigations are detailed in Table 1.

**Table 1:** Laboratory Investigations Performed on Arrival BUN: blood urea nitrogen; GGT: gamma glutamyl transferase; SGPT: serum glutamic pyruvic transaminase; AP: alkaline phosphatase; SGOT: serum glutamic oxaloacetic transaminase; LDH: lactate dehydrogenase; UA: uric acid; MCV: mean corpuscular volume; MCH: mean corpuscular haemoglobin; MCHC: mean corpuscular haemoglobin concentration; RDW: red cell distribution width; WBC: white blood cells; PT: prothrombin time; INR: international normalised ratio; APTT: activated partial thromboplastin time

Tests	Results	Units	Ranges
Magnesium	2.1	mg/dL	(1.6-2.6)
BUN	10	mg/dL	(6-20)
Creatinine	0.8	mg/dL	(0.6-1.1)
Sodium	141	mmol/L	(136-145)
Potassium	4.3	mmol/L	(3.5-5.1)
Chloride	110	mmol/L	(98-107)
Bicarbonate	21.0	mmol/L	(20-31)
Calcium	8.3	mg/dL	(8.6-10.2)
Total bilirubin	0.4	mg/dL	(0.1-1.2)
Direct bilirubin	0.3	mg/dL	(0-0.2)
Indirect bilirubin	0.1	mg/dL	(0.1-0.8)
GGT	81	IU/L	Female <38
SGPT	45	IU/L	Female <35
AP	343	IU/L	(45-129)
SGOT	41	IU/L	Female <31
LDH	312	IU/L	(135-214)
UA	6.2	mg/dL	(2.6-6)
Spot urine protein/creatinine ratio	75/32=2.3	Ratio	(<0.3)
Haemoglobin	10.7	g/dL	(11-14.5)
Hematocrit	35.2	%	(34.5-45.4)
Red blood cells	4.19	x10E12	(3.61-5.2)
MCV	84.0	fL	(78.1-95.3)
MCH	25.5	pg	(25.3-31.7)
MCHC	30.4	g/dL	(30.3-34.4)
RDW	17.0	%	(12.1-16.9)
WBC	7.7	x10E9/L	(4.6-10.8)
Neutrophils	49.5	%	(34.9-76.2)
Lymphocytes	42.7	%	(17.5-45)
Eosinophils	1.0	%	(0.3-7.4)
Monocytes	6.0	%	(3.9-10)
Basophils	0.8	%	(0-1)
Platelets	266	x10E9/L	(154-433)
PT	9.8	Seconds	(9.3-12.8)
INR	0.9	Ratio	(0.9-1.2)
APTT	30.6	Seconds	(22.9-34.5)

Labour room course

After the patient was moved to the labour room, her blood pressure remained persistently elevated. She was started on an intravenous labetalol infusion along with a maintenance dose of magnesium sulphate (1 gram/hour). Cardiotocography (CTG) revealed a pathological trace due to prolonged deceleration. A vaginal examination showed cervical dilation up to 4 cm, meconium-stained liquor, and a subsequent episode of variable decelerations on the CTG. Given the deteriorating maternal and fetal conditions, the decision was made to perform an emergency cesarean section. The patient was then transferred to the operating theatre for the procedure.

Operative findings

Due to the pathological trace, a category I cesarean section was performed. A Pfannenstiel incision was made on the skin, and entry into the peritoneal cavity was achieved by excising the subcutaneous fat, rectus sheath, muscle, and peritoneum. After opening the uterovesical fold, the bladder was reflected downward. A low transverse incision was made on the lower uterine segment, revealing that the fetal head was deeply impacted in the pelvis. Manual attempts to push the head upward through the vagina were unsuccessful. The cervix was found to be 7-8 cm dilated with a station at zero. An inverted T incision was made on the uterus to facilitate reverse breech extraction. The baby was delivered within seven minutes of the incision, weighing 1.5 kg, with an APGAR (Appearance, Pulse, Grimace, Activity and Respiration) score of 9 at 5 minutes. The placenta and membranes were completely removed.

**Figure 1:**
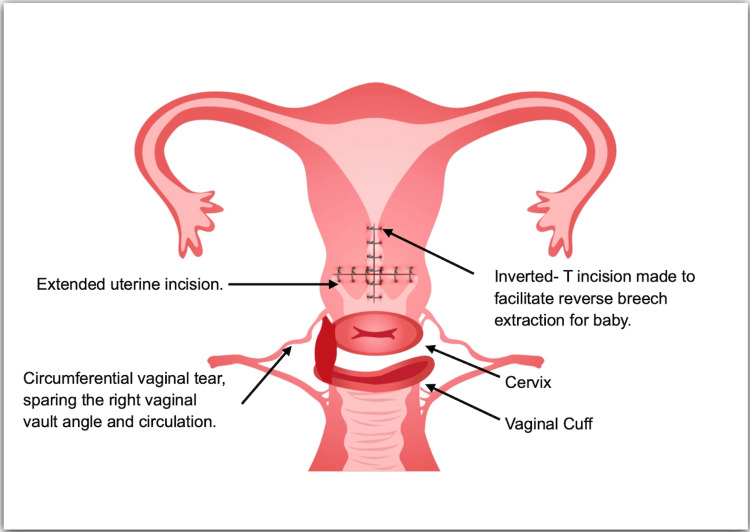
Diagrammatic Representation of Uterus and Vaginal Tear This figure has been drawn by the author using Adobe Illustrator (Adobe Inc., San Jose, USA).

After delivery, a full-thickness circumferential tear in the vaginal vault was observed, as shown in Figure 1. The right vaginal vault angle remained intact, and the bladder was undamaged. The integrity of the bladder was verified using methylene blue dye. Due to the complexity of the injury, urgent assistance was requested from a gynaecological oncology surgeon. The uterine cavity, with its low transverse and inverted T incisions, was closed in two layers with Vicryl 0 (Ethicon, Inc., Bridgewater, USA), and hemostasis was secured with Vicryl 2-0 after careful sharp dissection. The circumferential vaginal tear was sutured with Vicryl 2-0 using the same technique as in vaginal radical trachelectomy. Given the proximity of the left ureter to the vaginal cuff, the urology team was consulted to check for any urological injuries. The left ureter was traced from the mid-ureter to its entry into the bladder. Methylene blue dye was used in a retrograde manner, and no spillage was observed, indicating no iatrogenic injury to the ureter or bladder during the dissection. The patient was transfused with two units of packed red blood cells intraoperatively. The total duration of the surgery, from skin incision to dressing, was three hours and nine minutes, with an estimated blood loss of 1500 millilitres.

Postoperative course

Following surgery, the patient was placed in special care due to pre-eclampsia. She was administered broad-spectrum antibiotics (ceftriaxone and metronidazole), and maintenance doses of magnesium sulphate were continued for 24 hours. Her blood pressure remained within normal limits, and postoperative lab results were normal. On the evening of the first postoperative day, the patient experienced tachycardia and tachypnea, along with a fever spike to 38°C. Suspecting sepsis, lab investigations were conducted (as shown in Table 2) and antibiotics were escalated to meropenem and vancomycin. The patient responded well, and her condition improved. Within 24 hours of initiation of antibiotics, the leucocytosis improved (from 28.1 to 14.3x10^3/µL). She was transferred to general ward care on the second postoperative day. Her diet and activity levels were gradually increased, and she was discharged on the third day due to financial constraints. At the postnatal follow-up, both the patient and her baby were well, with no active complaints.

**Table 2:** Investigations Performed in Postoperative Course MCV: mean corpuscular volume; MCH: mean corpuscular haemoglobin; MCHC: mean corpuscular haemoglobin concentration; RDW: red cell distribution width; WBC: white blood cells

Tests	Results	Units	Ranges
Haemoglobin	8.1	g/dL	(11-14.5)
Hematocrit	24.5	%	(34.5-45.4)
Red blood cell	2.80	x10E12/L	(3.61-5.2)
MCV	87.5	fL	(78.1-95.3)
MCH	28.9	pg	(25.3-31.7)
MCHC	33.1	g/dL	(30.3-34.4)
RDW	15.2	%	(12.1-16.9)
WBC	28.1	x10E9/L	(4.6-10.8)
Neutrophils	87.2	%	(34.9-76.2)
Lymphocytes	9.6	%	(17.5-45)
Eosinophils	0.0	%	(0.3-7.4)
Monocytes	3.0	%	(3.9-10)
Basophils	0.2	%	(0-1)
Platelets	146	x10E9/L	(154-433)
C-reactive protein	228.00	mg/L	(0-14)

## Discussion

The management of obstetric emergencies, especially in unbooked patients with multiple risk factors, can be challenging. This case illustrates the complexity and highlights several important aspects worth discussing.

The patient presented with a history of pre-eclampsia and fetal growth restriction, which are well-known risk factors for adverse maternal and fetal outcomes. The need for a coordinated antenatal and perinatal management approach to mitigate these risks is evident. Pre-eclampsia, in particular, remains a leading cause of maternal morbidity and mortality worldwide. Early recognition, monitoring, and management are crucial to preventing severe complications [3,4]. The decision to proceed with an emergency cesarean section was justified due to the deteriorating maternal and fetal conditions. The deep impaction of the fetal head presented a challenge during the procedure. The inverted T incision on the uterus was necessary to facilitate the reverse breech extraction which highlights the importance of surgical expertise in dealing with complex cases [5,6].

This case documents an infrequent complication of circumferential vaginal tear arising during second-stage cesarean section. The surgical restoration of the normal anatomical structure successfully corrected the complete detachment of the uterus from the vaginal vault while preserving the uterine blood supply. This approach helped avert the need for peripartum hysterectomy, ultimately reducing the morbidity of the patient and preserving potential future reproductive capability. It is critical to promptly recognize and repair such injuries to prevent long-term consequences, including vaginal stenosis and urinary or faecal incontinence. The involvement of the urology team to assess for urological injuries was also a prudent approach.

A literature review showed a study conducted by Bergholt et al., aiming to identify the primary preoperative risk factors linked to intraoperative uterine tears during cesarean section. They identified that birth weight greater than 4000 g and a low fetal station or presenting part as the factors most strongly associated with this complication. The authors inferred that these elements likely add to the difficulty in delivering the fetus through the routine low transverse incision on the uterus [7]. Roughly 1.5% of cesarean deliveries encounter challenges with an impacted fetal head. Typically, this situation is addressed through either the 'push' technique, involving vaginal hand assistance, or the 'pull' method, which involves locating the baby's legs and performing a footling breech extraction. Veisi et al. discovered that patients undergoing the 'push' method experienced a higher incidence of uterine incision extensions compared to those undergoing the 'pull method' [8]. Similar findings were also concluded by Fasubaa et al. that the 'push' method is linked to greater rates of maternal morbidity including massive haemorrhage, increased chance of extension in incision on the uterus, frequent risks of endometritis, prolonged surgical duration, and extended hospital stays when compared with the 'pull' method [9]. With the 'push' method, the extension is ascribed to the force exerted by the vaginal hand, it is noteworthy that superior extensions of the uterus also occurred with ‘reverse breech extraction’ [8]. While research outlines the expansion of the uterine incision and acknowledges the potential force risk from the vaginal hand in the 'push' technique, there is no explicit literature reference to the formation of a distinct vaginal laceration with this method. However, it remains a plausible complication linked to the 'push' technique.

The physiological adaptations happening in the vaginal wall under the influence of pregnancy hormones could elucidate the occurrence of uterine detachment. A study conducted by Rahn et al. revealed that pregnancy results in an augmented resting diameter of the vaginal wall, enhanced tissue distensibility, and reduced strength in the vaginal wall collectively portraying the dynamic adaptations for the vaginal birth [10]. In a comprehensive sense, the physiologic changes induced by pregnancy affect the biomechanical properties of the vaginal wall, making it more vulnerable to damage when subjected to trauma or excessive stretching. In the 'push' method, the placement of the vaginal hand suggests that the upper portion of the vagina is prone to experiencing the most substantial physical strain during the manoeuvre. In such a scenario, a laceration with extension could result in the complete detachment of the uterus from the vaginal cuff.

Considering the patient's reproductive wishes, and especially since the uterine blood supply was preserved, there was a chance to reconnect the uterus and cervix to the vaginal cuff instead of resorting to peripartum hysterectomy. In this instance, a surgical approach akin to the one employed in fertility-sparing procedures for cervical cancer, specifically vaginal radical trachelectomy (VRT), was utilized. Although the patient's circumstances differed from VRT, as cervical transection was not necessary, the feasibility of this surgical repair was established through prior experience with VRT. Despite some impairment to the blood supply, the collateral blood flow to the pregnant uterus proved satisfactory. This form of repair not only addressed the immediate concern but also preserved the possibility of future fertility, eliminating the necessity for a postpartum hysterectomy.

The occurrence of complete detachment of the vaginal vault from the uterus and cervix is an uncommon complication linked to cesarean section, especially in cases where the fetus is deeply engaged in the vertex position and the 'push' technique with the vaginal hand is utilized. In instances where a patient prioritizes future fertility and the uterine blood supply remains intact with stable per-operative hemodynamics, it is advisable to consider attempting a repair. This case serves as an example, illustrating the practicality of a conservative treatment approach. This involves reattaching the vaginal cuff to restore anatomical integrity, presenting a viable alternative to opting for a cesarean hysterectomy [11]. Managing complications during cesarean sections requires a multidisciplinary approach involving obstetricians, neonatologists, anaesthesiologists, and, in this case, urologists. Close coordination among these specialists is crucial in ensuring the best outcomes for the mother and the newborn [12,13]. Postoperatively, the patient developed sepsis, underscoring the risk of infection following surgery. In this instance, the prolonged duration of surgery and the use of the 'push' method during an impacted fetal head delivery contributed to this complication. Utilizing broad-spectrum antibiotics appropriately is crucial for preventing and treating postoperative infections [14]. The patient's early discharge due to financial constraints reveals the economic challenges in healthcare access. This issue necessitates a broader discussion on healthcare equity and the provision of adequate financial support for patients in need [15]. The successful postoperative recovery of both the mother and the newborn is a positive outcome. It emphasizes the importance of timely involvement of multidisciplinary teams during surgery and comprehensive postnatal care to address any lingering concerns and ensure the well-being of both mother and baby.

## Conclusions

This case report highlights the critical importance of early diagnosis and prompt intervention in effectively managing complex obstetric cases. The occurrence of a circumferential vaginal tear during a cesarean section, while rare, serves as a poignant reminder of the necessity for vigilance in surgical practices and the collaboration of multiple specialities. The successful resolution of this case was achieved through the swift involvement of a gynaecological oncology surgeon and proactive consultation with the urology team. This multidisciplinary approach ensured comprehensive care and minimized the risk of further complications, demonstrating the advantages of involving various medical disciplines in managing intricate obstetric scenarios. For her next pregnancy, we recommend a preconception and early antenatal visit to an obstetrician to optimize her health and evaluate the risk for pre-eclampsia and fetal growth restriction, ensuring a planned cesarean delivery.
